# The defect-induced localization in many positions of the quantum random walk

**DOI:** 10.1038/srep25767

**Published:** 2016-05-24

**Authors:** Tian Chen, Xiangdong Zhang

**Affiliations:** 1 School of Physics, Beijing Institute of Technology, 100081, Beijing, China

## Abstract

We study the localization of probability distribution in a discrete quantum random walk on an infinite chain. With a phase defect introduced in any position of the quantum random walk (QRW), we have found that the localization of the probability distribution in the QRW emerges. Different localized behaviors of the probability distribution in the QRW are presented when the defect occupies different positions. Given that the coefficients of the localized stationary eigenstates relies on the coin operator, we reveal that when the defect occupies different positions, the amplitude of localized probability distribution in the QRW exhibits a non-trivial dependence on the coin operator.

The classical random walk (CRW) has proven to be a powerful technique in classical algorithms^1^. Its quantum counterpart, quantum random walk (QRW)^2^,^3^,^4^,^5^,^6^,^7^,^8^,^9^,^10^,^11^,^12^,^13^,^14^,^15^,^16^,^17^,^18^, has also been employed in developing some quantum algorithms, e.g., random-walk search algorithms^19^,^20^,^21^,^22^, quantum PageRank algorithms in a quantum network^23^,^24^,^25^, and so on. To design such quantum algorithms based on the QRW, it is necessary for us to explore and understand the properties of the QRW itself. One property associated with the efficient design of quantum algorithms is the localization of position distribution in the QRW^26^,^27^. The first paper devoted to the localization within quantum mechanics is presented by Anderson^28^. When the localization emerges in the QRW, the amplitude of probability distributions at some positions of the QRW keeps a nonzero value all the time, and the probability distribution in the position space does not show the ballistic spreading as in the standard QRW^3^. Several origins of the localization in the QRW have been discussed in detail^29^,^30^,^31^,^32^,^33^,^34^,^35^,^36^,^37^,^38^,^39^,^40^,^41^,^42^,^43^,^44^,^45^,^46^,^47^,^48^,^49^,^50^. When the entanglement is introduced into the coins or particles, or the multi-state coin is used, the localization in the QRW appears due to the emergence of the degeneracy of some eigenvalues for the evolution matrix *U*(*k*)^29^,^30^,^31^,^32^,^33^,^34^,^35^,^36^,^37^,^38^. When the QRW is affected by the random environment, the localization can be found in the QRW^39^,^40^,^41^,^42^,^43^,^44^,^45^,^46^,^47^. Another case for the appearance of the localization results from the inhomogeneity of the coin operators in the walk^48^,^49^,^50^. Moreover, the recurrence probability of the QRW has been analyzed and the criterion for the localization of the QRW has been presented^51^,^52^,^53^,^54^,^55^. Recently, some researches illustrated that when only the phase of the original position in the QRW is modified (it means that only one single phase defect is introduced at the original position), one will obtain a sharp allocation of distribution for this particular position and keep the amplitude of the localization until the infinite time^56^,^57^,^58^,^59^,^60^,^61^. This QRW incorporating one position-dependent phase defect has been realized with the aid of beam displacers and phase shifters in experiment already^62^,^63^.

In our work, we study the localization of the QRW on an infinite line in which the inhomogeneity is introduced. A phase defect appears in one position of the QRW. As stated in the previous paper^56^,^57^,^62^, when the defect occupies the position *x* = 0 or *x* = 1, the probability distribution at the corresponding position *x* = 0 or *x* = 1 in the QRW architecture will not tend to zero even the time approaches the infinite limit. Our results reveal that, when the defect is introduced into any position of the QRW, the localization of the probability distribution will appear. Given the localized stationary eigenstates of the step evolution operator obtained in Sec. *Methods*, we find that the amplitude of localized probability at the certain position of the QRW depends on the overlap between the localized stationary eigenstates of the step evolution operator and the initial state of the QRW. An interesting result is presented that when the defect occupies different positions, the amplitude of localized probability in the QRW reflects the non-trivial dependence on the parameter *θ* of the coin operator *C*(*θ*), not only shows a simple monotonic increase with the parameter *θ* as reported before^62^. Such property that the probability distribution of the QRW depends on the coin operator is very significant and has its application into the development of the quantum algorithms^23^,^24^,^25^,^26^,^27^. Besides, the effects of coin operator have been discussed in other aspects within the QRW, e.g., quantum state transfer, simulation of properties of nano-devices in spintronics, etc^64^,^65^,^66^. Based on the localized eigenstates of the step evolution operator provided in Sec. *Methods*, we present a reasonable analysis for the probability distribution in the QRW which shows a non-trivial dependence on the coin operator. A potential experimental realization of our QRW with the phase defect is proposed at the end of our main text.

The organization of our paper is as follows, in Sec. *Results*, we present the step evolution operator *U*_*ϕ*_ of the QRW with defect and in Subsec. *Localization with the defect occupying different positions*, we numerically obtain the position distribution of the QRW when the defect occupies different positions. Then we discuss two different QRWs that the defect resides at the even (*x* = 2) or odd (*x* = 3) position of the walk in Subsec. *The effect of coin operators on the localization*. With the localized stationary eigenstates of the step evolution operator presented in Sec. *Methods*, we analyze the effect of the coin operator on the position distribution of the QRW. A potential experimental realization for such QRW with defect is proposed. Later, we provide our conclusion and discuss the future application of our findings in Sec. *Discussion and Conclusion*.

## Results

The one step evolution operator in the QRW architecture is *U*_*ϕ*_ which consists of one coin operator *C*(*θ*) and one conditional shift operator 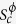.


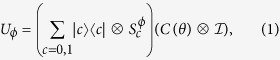


where the Hilbert space of coin operator 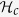 is spanned by |*c*〉, *c* = 0, 1, and the Hilbert space of position 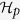 is spanned by |*x*〉, *x* ∈ ***Z***. The total system is comprised by the coin and the position. The coin operator *C*(*θ*) is *θ*-dependent, that is,


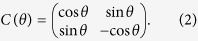


When *θ* = *π*/4, the coin operator takes the form as the familiar Hadamard matrix. The conditional shift operator 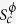 allows the particle to walk into two different directions according to the coin state,


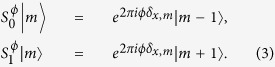


The effect of the defect is contained in the phase. When the particle walks past the position *x* = *m*, it will acquire an additional phase 2*πϕ*.

## Localization with the defect occupying different positions

**Figure 1:**
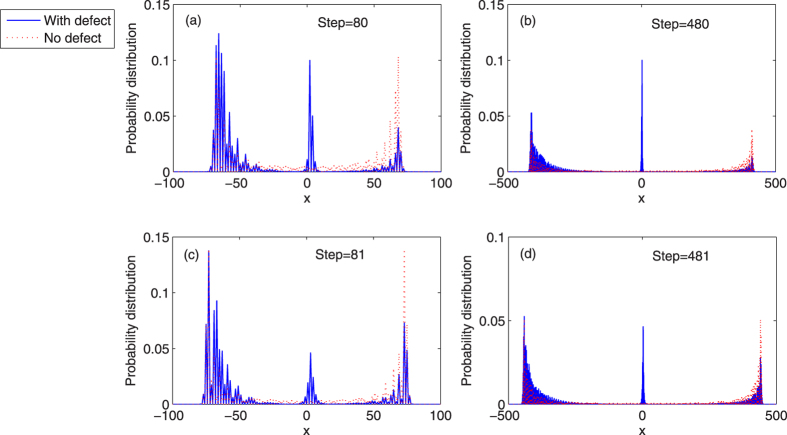
The probability distribution of the position in the QRW with different steps. Blue solid, the probability distribution of the QRW with one single phase defect. Red dotted, the probability distribution of the standard QRW without defect. The initial state of the coin and position is taken as 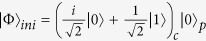. The phase of the defect, *ϕ* = 1/2. (**a**,**b**) the defect occupies the position *x* = 2. A sharp peak of probability is found at the position *x* = 2. The parameter *θ* of the coin operator is chosen, *θ* = *π*/6. (**c**,**d**) the defect occupies the position *x* = 3. A sharp peak of probability is found at the position *x* = 3. The parameter *θ* of the coin operator is chosen, *θ* = *π*/8.

In this subsection, we numerically study the localized probability at different positions in the QRW. As stated previously^56^, the localization of the probability distribution in the QRW means that the amplitude of probability at certain position will keep a non-zero value all the time. It is well known that, if the particle starts from the original position (*x* = 0), the particle will occupy only even (odd) positions with the even (odd) step evolution. In our numerical calculation, when the defect occupies the position *x* = 0 or *x* = 1, we can reproduce the same probability distributions of the QRW with or without defects as reported in refs 56, 57, 58. Then we explore the properties of probability distribution of the QRW in which the defect occupies a farther position (*x* ≥ 2). We take the single phase defect locating at the position *x* = 2 or *x* = 3 as examples. The initial state for the coin and the position is 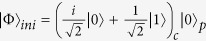. The phase of the defect is *ϕ* = 1/2. The particle starts from the original point *x* = 0, then undergoes many steps of the evolution in the quantum walk architecture with the single phase defect occupying the position *x* = 2 or *x* = 3. The probability distributions of the QRW with and without defects are presented in Fig. 1.

It is clearly seen that when the defect appears at the position *x* = 2 (Fig. 1(a,b)) or *x* = 3 (Fig. 1(c,d)), the probability of occupying the position around *x* = 2 (Fig. 1(a,b)) or *x* = 3 (Fig. 1(c,d)) does not tend to zero, no matter how many steps the particle has taken (Blue solid lines in Fig. 1). The localized probability at the position *x* = 2 or *x* = 3 keeps the same value with the increase of steps. While, the probability distribution of the standard QRW without defects shows the ballistic spreading. No localization of probability distribution in the QRW can be found in such case (Red dotted lines in Fig. 1). For the QRW with single phase defect, the localization of the position distribution results from the emergence of the localized eigenstates of the step evolution operator 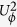. The initial state of the QRW evolves with the unitary step evolution operator *U*_*ϕ*_. If there is non-zero overlap between the initial state of the QRW and the localized eigenstates of 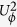, some of the initial state will evolve into the localized eigenstates of 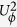 and keep unchanged with the evolution. Then the localization of the QRW emerges. As presented in Sec. *Methods*, when the defect occupies the position *x* = *m*, we can obtain the eigenstates of 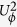 as |*ψ*〉 = ∑_*n*_(*α*_*n*_|0〉_*c*_|*n*〉_*p*_ + *β*_*n*_|1〉_*c*_|*n*〉_*p*_), the expression of the coefficients of *α*_*n*_ and *β*_*n*_ can be explicitly written as

*when n* ≠ m,


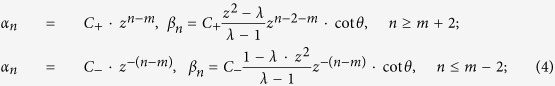


*when n* = m;


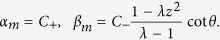


**Figure 2:**
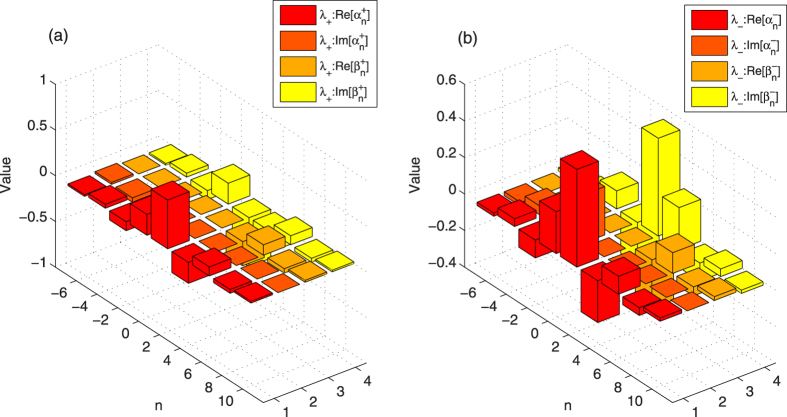
The coefficients 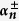 and 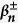 of the localized eigenstates |*ψ*_±_〉 of the step evolution operator 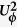. The position of the defect is *x* = *m* = 2, the parameter *θ* of the coin operator is *θ* = *π*/6; the phase defect *ϕ* = 1/2. In (**a**), the cuboid with red and orange denotes the real and imaginary part of 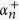, the cuboid with dark yellow and light yellow represents the real and imaginary part of 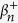; In (**b**), the cuboid with red and orange denotes the real and imaginary part of 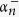, the cuboid with dark yellow and light yellow represents the real and imaginary part of 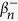.

Where *λ* is the eigenvalue of the operator 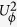, the constants *C*_+_ and *C*_−_ have a relation shown in Sec. *Methods*. After taking the normalization for the eigenstate |*ψ*〉, we can obtain the coefficients *α*_*n*_ and *β*_*n*_ of the eigenstates |*ψ*〉 of the QRW. Here, we take the defect occupying the position *x* = *m* = 2 as an example, the parameter *θ* for the coin operator is *θ* = *π*/6, the phase of the defect is *ϕ* = 1/2. Following the method addressed in Sec. *Methods*, we can obtain two different eigenvalues *λ*_±_ of evolution operator 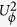. The detailed description of the localized eigenstates |*ψ*_+_〉 (|*ψ*_−_〉) of the evolution operator 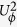 corresponding to the eigenvalue *λ*_+_ (*λ*_−_) is presented in Fig. 2.

The left part of Fig. 2 describes the coefficients 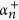 and 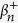 of localized eigenstates 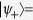 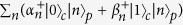 with the corresponding eigenvalue *λ*_ + _, and the details of 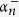 and 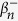 of the localized eigenstates 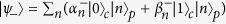 are presented in the right part of Fig. 2. Due to the emergence of localized eigenstates |*ψ*_±_〉 of the step evolution operator 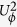 in the QRW, if the overlap between the initial states |Φ〉_*ini*_ and the localized eigenstates |*ψ*_±_〉 is not zero, the localization in the QRW can appear. Considering the case addressed in Fig. 1(a,b), when the initial state of the QRW is 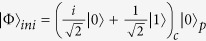, the defect occupies the position *x* = 2, the parameter *θ* of the coin operator is *π*/6, the overlap between the localized eigenstate |*ψ*_+_〉(|*ψ*_−_〉) and the initial state |Φ〉_*ini*_ is 0.03499 (0.1399), so the localization of the QRW appear. Though in small steps of evolution (Fig. 1(a,c)), the localized probability at the position *x* = 2 or *x* = 3 in the QRW mingles with the diffusion of the probability, the localization becomes apparent when the step is large (Fig. 1(b,d)). Moreover, the “three-peak-zones” of the position distribution emerges in the QRW with defect, which is similar as the position distribution of QRW with entangled coins^32^,^33^,^34^,^35^. For the QRW with entangled coins, the reason for the localization around the starting point is explained as the emergence of the degeneracy of some eigenvalues for the evolution matrix *U*(*k*)^34^,^35^. In their discussions, the evolution matrix *U*(*k*) is the Fourier transform of the step evolution operator. For our studied QRW with defect, the localization of position distribution results from the appearance of the localized eigenstates of the step evolution operator 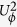 and the non-zero overlap between the localized eigenstates and the initial states of the QRW.

Another interesting feature is that the QRW with defects exhibits an asymmetrical distribution around the defect’s position *x* = 2 (Fig. 1(a,b)). Due to the reflection of the defect, a larger probability distribution can be found in the left side of the position *x* = 2, when compared to the smaller probability of transmission in the right region of the position *x* = 2 58,59. The similar behaviors of probability distribution can be found when the defect occupies the position *x* = 3, see Fig. 1(c,d).

What’s more, we study the localization of probability distribution in the QRW when the defect occupies different positions. The particle starts from the original position (*x* = 0), and the initial state for the coin and the position is 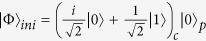. The phase of the defect is *ϕ* = 1/2. The parameter *θ* of the coin operator is set as *θ* = *π*/6.

**Figure 3:**
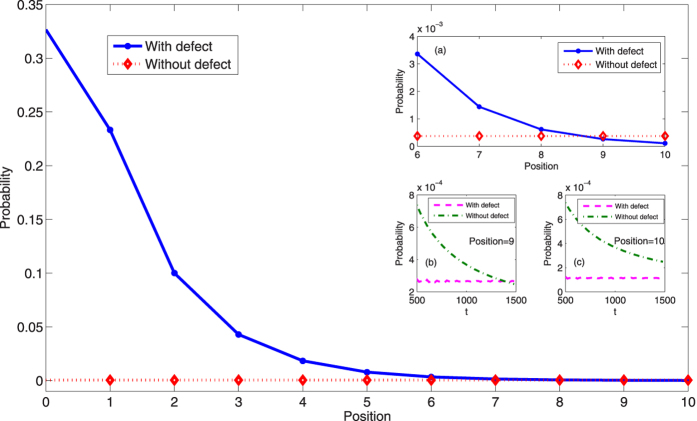
The amplitude of localized probability at the position where the defect occupies. For the defect resides at the even positions, the step of the evolution in the QRW is 980; for the defect occupies the odd positions, the step of the evolution in the QRW is 981. The initial state of the coin and position is taken as 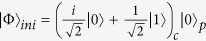. The phase of the defect *ϕ* = 1/2; the coin operator with *θ* = *π*/6. Blue solid, the QRW with one defect; red dotted, the QRW without defects. In inset (**a**), the defect changes from the position *x* = 6 to *x* = 10, and the localized probability at the corresponding position is addressed. The inset (**b**,**c**) describe the time evolution of probability at the position *x* = 9 and *x* = 10, respectively. Inset (**b**), the purple dashed line represents the localized probability at the position *x* = 9 and the defect occupies the same position *x* = 9; the dotted dashed green line denotes the time evolution of probability at *x* = 9 without defect. Inset (**c**), the purple dashed line stands for the localized probability at *x* = 10 and the defect is at the same position *x* = 10; the dotted dashed green line represents the time evolution of probability at *x* = 10 without defect.

In Fig. 3, we study the localized probability at different positions where the defect occupies. The blue solid line represents the localized probability at the certain position in the QRW and the defect occupies the same position. The red dotted line denotes the probability at the certain position in the QRW without defect. For the defect resides at the even positions, the step of the evolution in the QRW is 980; for the defect occupies the odd position, the step of the evolution in the QRW is 981. Though the magnitude of probability localized at the position *x* ≥ 6 is small, such probability will never decrease to zero with increasing the step evolution of the QRW. The detailed description of the amplitude of the probability distribution for the position *x* ≥ 6 is presented in the inset (a) of Fig. 3. We find that when the step of the evolution is around 1000, the probability at the position *x* = 9 or *x* = 10 of the QRW with defect is smaller than that of standard QRW without defects. The time evolution of the QRW with or without defect is explicitly provided in insets (b) and (c) of Fig. 3. In the inset (b,c), we discuss the probability of *x* = 9 (*x* = 10) in the QRW with time. The purple dashed line describes the time evolution of the probability of *x* = 9 (*x* = 10) in the QRW with defect, and in comparison, the green dotted dashed line represents the time evolution of the probability of *x* = 9 (*x* = 10) in the standard QRW without defect. From these two insets, we find that when the defect occupies the position *x* = 9 (*x* = 10), the amplitude of probability distribution at the corresponding position *x* = 9 (*x* = 10) keeps around the same value with time evolution of the QRW (see the purple dashed lines in the insets (b) and (c), the time for the QRW is from 500 to 1500). While, in the standard QRW without defect, the probability at the position *x* = 9 and *x* = 10 decays exponentially with the time (see the green dotted dashed lines in the insets (b) and (c))^2^,^3^,^8^. It indicates that when the phase defect exists in the QRW, the localization of the probability distribution in the QRW will appear.

## The effect of coin operators on the localization

In this subsection, we study the effect of different coin operators on the localization in the QRW. Considering the step evolution operator 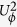 contains two steps evolution in the QRW, at first, we explore the properties of the probability distribution in the QRW with the defect occupying the even position, then the QRW with the defect occupying the odd position is discussed.

### Even case

**Figure 4:**
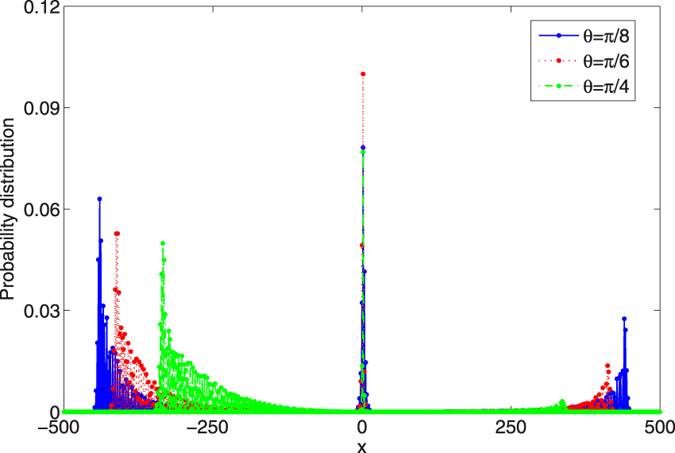
The probability distribution of the position in the QRW with the defect residing at *x* = 2. Three different *θ*s of the coin operators are chosen. Blue solid, *θ* = *π*/8, red dotted, *θ* = *π*/6, green dotted dashed, *θ* = *π*/4. The initial state of the coin and position is taken as 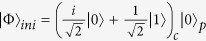. The phase of the defect *ϕ* = 1/2. The time step of the evolution in the QRW is 480.

To study the QRW with the defect occupying the even position, we take the QRW with defect occupying the position *x* = 2 as an example. The particle starts from the original position (*x* = 0), and then the particle undergoes the even steps evolution. The initial state for the QRW is 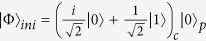. The phase of the defect is *ϕ* = 1/2. In our discussion, we take three different values for the parameter *θ* of the coin operator, that is *θ* = *π*/8, *π*/6 and *π*/4. The time step of the evolution in the QRW is 480. The probability distributions of the QRW with these three different coin operators are addressed in Fig. 4.

From the figure, we can find that among the parameter *θ* = *π*/8, *π*/6, and *π*/4 of the coin operator, when *θ* is taken as *π*/6, the localized probability at the position *x* = 2 where the defect occupies is largest. While in comparison, when the phase defect emerges at the position *x* = 0 (*x* = 1), the localization of the probability distribution in the QRW can be found at the position *x* = 0 (*x* = 1), the amplitude of the localized probability displays the monotonic increase with the parameter *θ* of the coin operator^62^.

**Figure 5:**
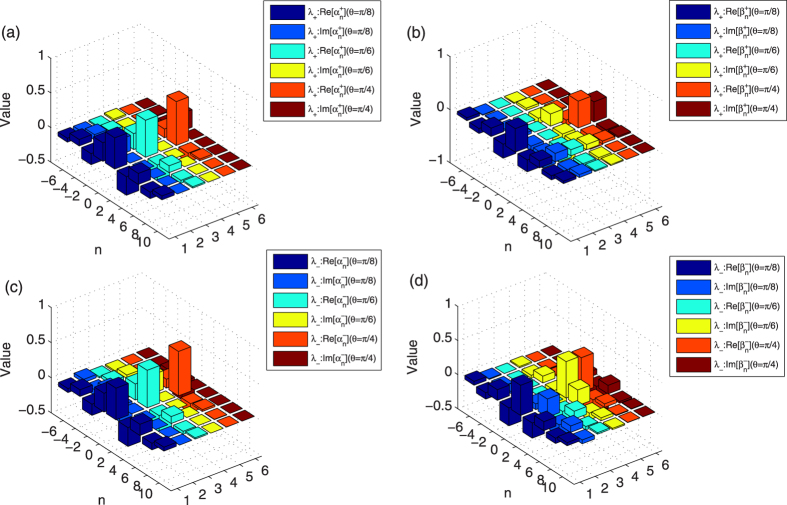
The values 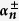 and 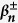 of the localized eigenstates |*ψ*_±_〉 of step evolution operator 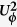. The defect occupies *x* = 2. The phase of the defect *ϕ* = 1/2. (**a**,**b**) describe the coefficients 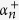 and 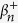 for the eigenstate |*ψ*_+_〉. In figure (**a**,**b**), the cuboid with dark blue and medium blue denotes the real and imaginary part of 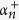 (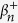) with *θ* = *π*/8; the cuboid with cyan and yellow denotes the real and imaginary part of 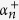 (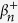) with *θ* = *π*/6; the cuboid with orange and crimson denotes the real and imaginary part of 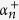 (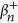) with *θ* = *π*/4. (**c**,**d**) describe the coefficients 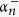 and 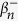 for the eigenstate |*ψ*_−_〉. In figure (**c**,**d**), the cuboid with dark blue and medium blue denotes the real and imaginary part of 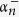 (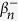) with *θ* = *π*/8; the cuboid with cyan and yellow denotes the real and imaginary part of 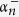 (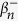) with *θ* = *π*/6; the cuboid with orange and crimson denotes the real and imaginary part of 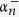 (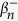) with *θ* = *π*/4.

Now, for the defect occupies the position *x* = 2, we begin to analyze the amplitude of localized probability that is the non-monotonic increase with *θ* in the QRW. The phase of the defect is *ϕ* = 1/2. For each value of these three different *θ*s (*θ* = *π*/8, *π*/6, and *π*/4), by applying the calculation methods in Sec. *Methods*, we obtain two different eigenvalues (*λ*_+_ and *λ*_−_) and the corresponding eigenstates 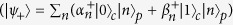 and 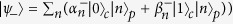 of the step evolution operator 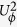. The coefficients 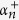 and 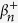 (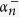 and 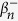) of the localized eigenstates |*ψ*_+_〉 (|*ψ*_−_〉) are presented in Fig. 5. The figures (a) and (b) of Fig. 5 describe the coefficients 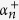 and 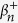, respectively. In figure (a,b)), the cuboid with dark blue and medium blue denotes the real and imaginary part of 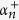 (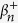) with the parameter *θ* chosen as *θ* = *π*/8; the cuboid with cyan and yellow denotes the real and imaginary part of 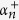 (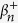) with *θ* = *π*/6; the cuboid with orange and crimson denotes the real and imaginary part of 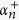 (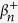) with *θ* = *π*/4. The figures (c) and (d) of Fig. 5 describe the coefficients 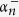 and 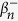 for the eigenstate |*ψ*_−_〉, respectively. In figure (c,d)), the cuboid with dark blue and medium blue denotes the real and imaginary part of 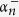 (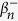) with the parameter *θ* chosen as *θ* = *π*/8; the cuboid with cyan and yellow denotes the real and imaginary part of 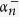 (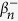) with *θ* = *π*/6; the cuboid with orange and crimson denotes the real and imaginary part of 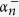 (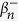) with *θ* = *π*/4. In our discussion, when the overlap between the localized eigenstates of the step evolution operator 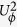 and the initial state |Φ〉_*ini*_ = (cos *φ* · *e*^*iδ*^|0〉 + sin *φ*|1〉)_*c*_|0〉_*p*_ is not zero, we will obtain the localized probability distribution in the QRW. For the defect occupies the even position *x* = *m*, the localized probabilities of the particle at position *x* = *l* in the QRW with respect to different localized eigenstates |*ψ*_+_〉 and |*ψ*_−_〉 are


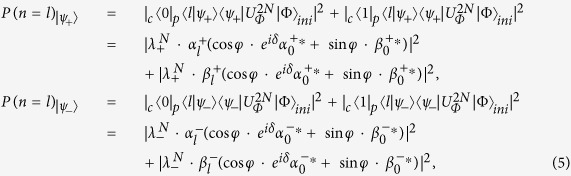


where the superscript 2*N* of the operator *U* implies that the particle of the QRW takes *N* evolution operator 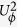. In our depiction of Fig. 4, the parameters of our initial state |Φ〉_*ini*_ is taken as *φ* = *π*/4 and *δ* = *π*/2. The phase of the defect is *ϕ* = 1/2. The localized probability at the position *x* = 2 of Fig. 4 corresponds to the parameter *l* = 2 in the expression of equation (5) above. The amplitude of localized probability with respect to the eigenstate *λ*_+_ (*λ*_−_) is associated with the coefficients 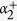, 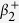, 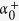 and 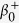 (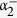, 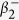, 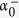 and 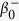). For the localized probability with the eigenstate |*ψ*_+_〉 (|*ψ*_−_〉), it indicates that the localized probability at the position *x* = 2 is not only related to the modulus of 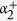 and 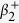 (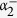 and 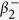), but also related to the real and imaginary part of 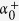 and 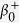 (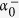 and 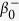). As shown in Fig. 5, though the modulus of 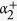 and 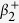 (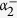 and 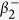) become larger with the increase of the parameter *θ* from *π*/8 to *π*/4, the real and imaginary part of 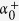 and 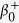 (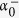 and 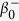) do not show the similar behavior. By applying the obtained coefficients 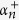 and 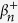 (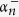 and 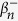) of the localized eigenstate |*ψ*_+_〉 (|*ψ*_−_〉) (see Fig. 5), we can make the sum of localized probabilities from the localized eigenstates |*ψ*_+_〉 and |*ψ*_−_〉, and obtain the amplitude of localized probability at position *x* = 2 in the QRW. With the initial state of the QRW 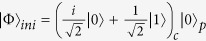, our calculation reveals the amplitude of localized probabilities as 0.07824, 0.09996 and 0.07680 with the parameter *θ* of the coin operator chosen as *π*/8, *π*/6 and *π*/4, respectively. In comparison, our numerical simulation of the evolution of the QRW provides the probability distribution of the QRW at time step 480 (see Fig. 4), the localized probabilities at the position *x* = 2 in the QRW are 0.07817, 0.09997 and 0.07679 with the parameter *θ* of the coin operator is *π*/8, *π*/6 and *π*/4, respectively.

### Odd case

**Figure 6:**
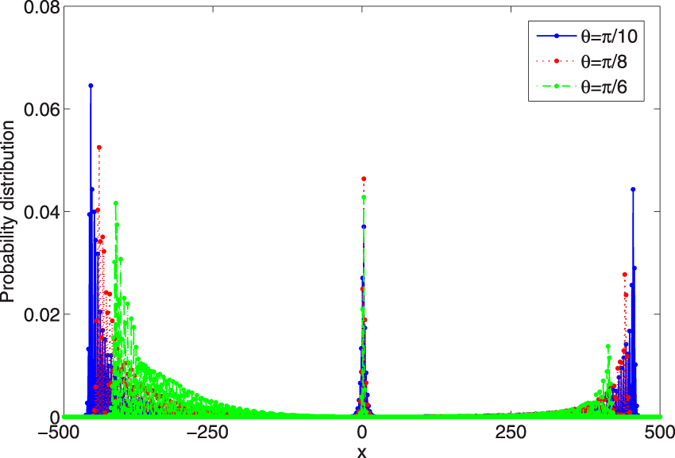
The probability distribution of the position in the QRW with the defect residing at *x* = 3. Three different *θ*s of the coin operators are chosen. Blue solid, *θ* = *π*/10, red dotted, *θ* = *π*/8, green dotted dashed, *θ* = *π*/6. The initial state of the coin and position is taken as 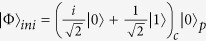. The phase of the defect *ϕ* = 1/2. The time step of the evolution in the QRW is 481.

For the defect appears at the even position, we have taken the defect occupying the position *x* = 2 as the example. Next, we will consider the probability distribution of the QRW with the defect occupying the odd position. The position *x* = 3 is chosen as the location of the phase defect. The particle starts from the original position, *x* = 0. The initial state of the coin and the position is 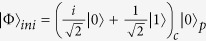. The phase defect *ϕ* = 1/2. In our discussion, three different coin operators are chosen as *θ* = *π*/10, *π*/8, and *π*/6. The time step of the evolution in the QRW is 481. The amplitudes of the probability distribution in the QRW are revealed in Fig. 6.

As shown in Fig. 6, the amplitude of localized probability at the position *x* = 3 does not increase monotonically with the parameter *θ* of the coin operator. The probability at the position *x* = 3 with *θ* = *π*/8 is larger than the probability at the same position with *θ* = *π*/10 or *θ* = *π*/6. While, the localized probability with the defect at the position *x* = 0 or *x* = 1 shows the monotonic increase with *θ*^62^. In the following, we will analyze the amplitude of localized probability at the position *x* = 3 in the QRW with different *θ*s of the coin operator.

**Figure 7:**
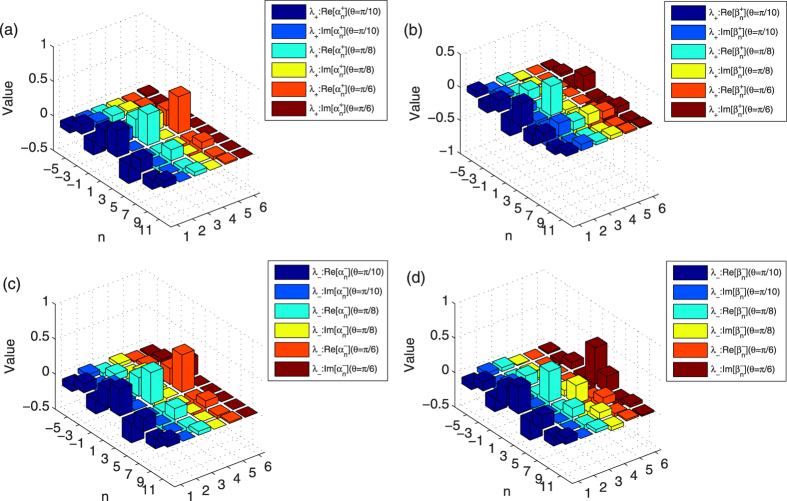
The values 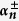 and 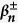 of the localized eigenstates |*ψ*_±_〉 of step evolution operator 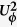. The defect occupies *x* = 3. The phase of the defect *ϕ* = 1/2. (**a**,**b**) describe the coefficients 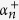 and 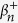 for the eigenstate |*ψ*_+_〉. In figure (**a**,**b**), the cuboid with dark blue and medium blue denotes the real and imaginary part of 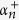 (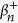) with *θ* = *π*/10; the cuboid with cyan and yellow denotes the real and imaginary part of 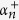 (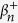) with *θ* = *π*/8; the cuboid with orange and crimson denotes the real and imaginary part of 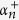 (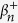) with *θ* = *π*/6. (**c**,**d**) describe the coefficients 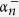 and 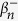 for the eigenstate |*ψ*_−_〉. In figure (**c**,**d**), the cuboid with dark blue and medium blue denotes the real and imaginary part of 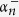 (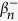) with *θ* = *π*/10; the cuboid with cyan and yellow denotes the real and imaginary part of 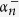 (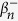) with *θ* = *π*/8; the cuboid with orange and crimson denotes the real and imaginary part of 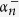 (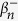) with *θ* = *π*/6.

We start by studying the localized eigenstates of the step evolution operator 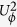. The phase of the defect is *ϕ* = 1/2. Considering three different *θ*s (*θ* = *π*/10, *π*/8 and *π*/6) of the coin operator, we can obtain two eigenvalues (*λ*_+_ and *λ*_−_) and two localized eigenstates 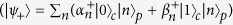 and 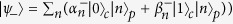 for the evolution operator 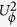 with each *θ*. The detailed description of the localized eigenstates for these three *θ*s is presented in Fig. 7. In Fig. 7(a,b), the cuboid with dark blue and medium blue denotes the real and imaginary part of 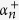 (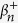) with *θ* = *π*/10; the cuboid with cyan and yellow represents the real and imaginary part of 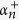 (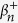) with *θ* = *π*/8; the cuboid with orange and crimson stands for the real and imaginary part of 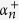 (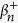) with *θ* = *π*/6. For Fig. 7(c,d), the cuboid with dark blue and medium blue corresponds to the real and imaginary part of 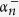 (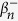) with *θ* = *π*/10; the cuboid with cyan and yellow represents the real and imaginary part of 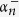 (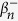) with *θ* = *π*/8; the cuboid with orange and crimson denotes the real and imaginary part of 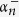 (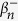) with *θ* = *π*/6. The amplitude of localized probability in the QRW depends on the overlap between the localized eigenstates of the step evolution operator 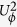 and the initial state of the QRW. Considering the step evolution operator 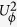 contains two steps evolution of the QRW, and the initial state of the QRW is expressed as |Φ〉_*ini*_ = (cos *φ* · *e*^*iδ*^|0〉 + sin *φ*|1〉)_*c*_|0〉_*p*_, we can obtain the state of the coin and the position after the first step as


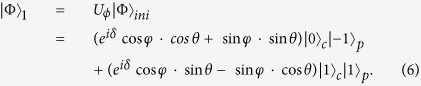


Then the localized probabilities at position *x* = *l* in the QRW with respect to different localized eigenstates (|*ψ*_+_〉 and |*ψ*_−_〉) can be addressed as


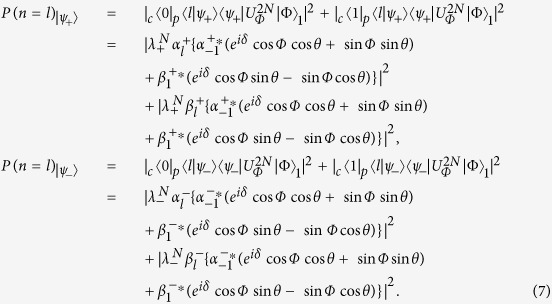


The coefficients for the localized probability at the position *x* = *l* = 3 corresponding to the localized eigenstate |*ψ*_+_〉 (|*ψ*_−_〉) are 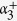, 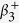, 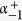 and 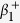 (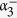, 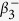, 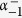 and 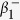).

When considering the localized probability contributed from the eigenstate |*ψ*_+_〉 (|*ψ*_−_〉), it not only depends on the modulus of 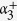 and 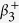 (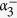 and 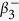), but also relates to the real and imaginary part of 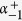 and 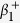 (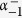 and 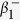). As shown in Fig. 7, though the modulus of 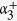 and 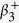 (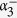 and 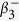) become larger with the increase of *θ*, the real and imaginary part of 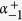 and 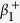 (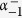 and 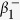) do not have the similar behavior. Actually from Fig. 7, we can find that the amplitudes of real and imaginary part of 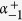 or 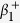 (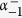 or 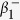) decrease with the change of *θ* from *π*/10, *π*/8 to *π*/6. By employing the coefficients obtained in Fig. 7, we calculate the localized probability of the QRW with the equation (7). With the initial state of the QRW 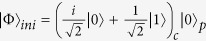, our results reveal that the localized probabilities at the position *x* = 3 in the QRW are 0.03711, 0.04639 and 0.04284 corresponding to the parameter *θ* = *π*/10, *π*/8 and *π*/6 of the coin operator, respectively. These values are similar to the localized probabilities at the position *x* = 3 from the numerical simulation of the QRW in Fig. 6, where the amplitudes of localized probabilities are 0.03706, 0.04637 and 0.04283 with *θ* = *π*/10, *π*/8 and *π*/6 of the coin operator, respectively.

Based on the discussion above, we have found that when there exists one phase defect at any position in the QRW, due to the non-zero overlap between the localized eigenstates of the step evolution operator and the initial state of the QRW, the localization of the probability distribution in the QRW appear. When the defect occupies at different positions, the amplitudes of localized probability in the QRW reveal different dependence on the coin operator. Our analysis on the localized probability above takes the position *x* = 2 and *x* = 3 as the defect’s position, similar analysis on the localization of the QRW can be discussed when the defect occupies the position *x* ≥ 4.

So far, we have studied theoretically the localization of probability distribution in the QRW with defects. Such localization of probability distribution can be observed in the experiment as realized in refs 15 and 16. In their experiments, the Hilbert space for the coin operator is spanned by the polarization degree of the light, and the step evolution is realized with the polarizing beam splitters (PBS) and fiber lines. Different positions in the QRW is revealed with different arriving times of photons in the avalanche photodiodes (APD). By applying the time-dependent signal to the electro-optic modulator (EOM), the phase defect can be introduced into the certain position of the QRW. Considering the QRW with the defect occupying the position *x* = 2, we find that the localization of probability distribution is apparent when the particle undergoes 30 steps evolution in the QRW. For the experimental realization mentioned above, the standard QRW with 28 steps evolution has been achieved^15^. This experimental realization might provide a platform to observe the localization of the probability distribution in the QRW with defects.

## Discussion and Conclusion

In summary, we have studied the localization of the position distribution in the QRW on an infinite chain. When the single phase defect is introduced into any position of the QRW, the probability at that position where the defect occupies does not tend to zero in the infinite time limit, and the localization of the probability distribution in the QRW emerges. Later we discuss the effect of different coin operators on the localization of the QRW. When the defect occupies different positions, the amplitudes of localized probability show the different dependence on the coin operator. Taking the defect residing at the position *x* = 2 or *x* = 3 as examples, we find that the localized probability at the position *x* = 2 or *x* = 3 does not go up monotonically with the increase of *θ*. Such non-trivial *θ*-dependence of localized probability in the QRW is different from that when the defect locates at the position *x* = 0 or *x* = 1, in which a trivial monotonic increase of localized probability with *θ* is revealed^2^,^3^,^62^. So the *θ* corresponding to the largest localized probability at the position *x* = 2 or *x* = 3 is not simply *π*/2. Further analysis on the localization when the defect resides at the position *x* ≥ 4 can be addressed in a similar way. Considering the goal of quantum algorithm is to find a specified vertex on the line with a probability of *O*(1), by introducing the defect into such specified vertex on the line, we can obtain a large probability of occupying the defect’s position with an appropriate choice of coin operator. Our new findings of localization in the QRW with defects not only deepen our insight into the properties of the QRW, but also help us to design quantum algorithms based on the QRW.

## Methods

In this section, we will provide the detailed derivation for the localized eigenvalues and eigenstates of the step evolution operator 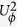. We assume that the state comprising the position and the coin is


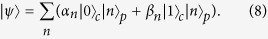


The subscript *c* (*p*) indicates that this state belongs to the Hilbert space for the coin (position). The phase defect occupies the position *x* = *m*. After applying one step *U*_*ϕ*_ to the total system, we obtain the expressions of the amplitude *α*_*n*_ and *β*_*n*_ when the particle starts from the position *x* = *n* = *m* at the discrete time *t*,


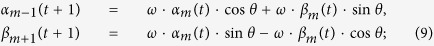


Here, the parameter *ω* denotes the phase *e*^2*πiϕ*^, with *ϕ* ∈ [0, 1). When the particle starts from the position *n* ≠ *m* at time *t*, the time evolution for coefficients *α*_*n*_ and *β*_*n*_ are


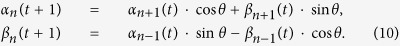


Considering the particle starts from the original position (*x* = 0) initially, it is clearly that the particle occupies even (odd) positions in the QRW architecture when the particle takes the even (odd) steps. To find the localized stationary states of the QRW with the defect, we apply two steps evolution operator 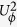 for the total system. We provide the relation for the coefficients at the position *x* = *n* between the time *t* and *t* + 2, and then derive the relation among the coefficients of localized eigenstates as in ref. 56. The probability amplitude *α*_*n*_ and *β*_*n*_ (*n* ≠ *m*) can be obtained as


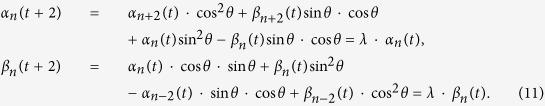


Here, the parameter *λ* stands for the eigenvalues of 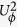. With equation (11), we can get


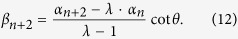


Substituting the expression of *β*_*n*_ into equation (11), we achieve the expression as


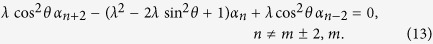


The general solution of this equation is


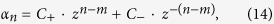


where *C*_+_ and *C*_−_ are constant coefficients. Considering the convergence of *α*_*n*_ when *n* → ±∞, we can obtain the expression for *α*_*n*_ with substituting equation (14) into equation (13),


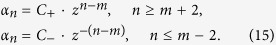


Here, *z* is the solution of equation (13) when its value satisfies, |*z*| < 1. With replacing the expressions of *α*_*n*_ above into into equation (12), we can obtain *β*_*n*_ as


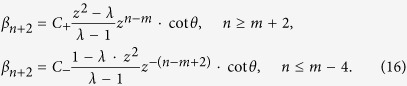


Taking into account the additional phase acquired when the particle walks through the defect at position *x* = *m*, we can get the coupled equations for the probability amplitude *α*_*n*_ and *β*_*n*_ (*n* = *m*) with the evolution operator 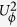 as


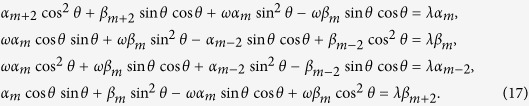


The parameter *λ* stands for the eigenvalues of 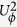, also. Following the obtained equations above, the explicit expressions for *α*_*m*_ and *β*_*m*_ are


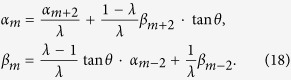


With the aid of representations as, *α*_*m*+2_ = *C*_+_ · *z*^2^, *α*_*m*−2_ = *C*_−_ · *z*^2^, 
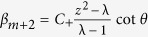
 , and 
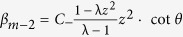
 , we obtain the probability distribution *α*_*m*_ and *β*_*m*_ at the position *x* = *m* that the defect occupies as


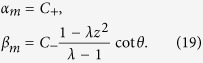


We replace the term *α*_*n*_ and *β*_*n*_ of equation (17) with the expressions above. The relation between the phase *ω* induced by the defect, the eigenvalue *λ* of 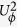 and the angle *θ* of the coin operator is shown as


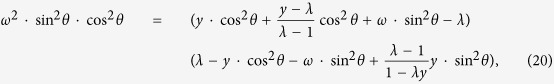


where we use *y* to replace *z*^2^. The expression for *y* can be obtained as


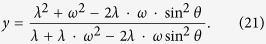


The relation between the constants *C*_+_ and *C*_−_ is


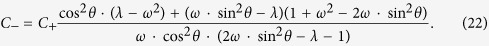


Considering the normalized condition for the summation of |*α*_*n*_|^2^ and |*β*_*n*_|^2^, we can get the values of *C*_+_ and *C*_−_, and the coefficients *α*_*n*_ and *β*_*n*_ at different positions *n* can be obtained.

## Additional Information

**How to cite this article**: Chen, T. and Zhang, X. The defect-induced localization in many positions of the quantum random walk. *Sci. Rep*. **6**, 25767; doi: 10.1038/srep25767 (2016).
